# Aerodynamic effects of dimples on soccer ball surfaces

**DOI:** 10.1016/j.heliyon.2017.e00432

**Published:** 2017-10-31

**Authors:** Sungchan Hong, Takeshi Asai

**Affiliations:** a Advanced Research Initiative for Human High Performance (ARIHHP), University of Tsukuba, Tsukuba, 305-8574, Japan; b Institute of Health and Sports Science, University of Tsukuba, Tsukuba 305-8574, Japan

**Keywords:** Engineering, Mechanics

## Abstract

Recently, the shape and design of the panel on the official ball used in the FIFA World Cup was considerably different from that of a conventional soccer ball (having 32 pentagonal and hexagonal panels). Depending on the number of different panels and their orientation, the aerodynamic force experienced by a ball is believed to change, which in turn changes the ball trajectory. However, not much is known about the impact of the surface forms of a ball on its aerodynamics. Therefore, in the present study, 10 different types of soccer balls were produced and their aerodynamic properties were studied by wind tunnel experiments. The results confirmed that the aerodynamic force acting on the ball varied considerably depending on the existence of dimples on the ball surface. In addition, the 4 types of soccer balls, which had different kinds of roughness, revealed that even balls having the same number and shapes of panels experienced greatly varying aerodynamic forces depending on the surface form of the balls.

## 1 Introduction

The pattern of a modern soccer ball is greatly different from that of a conventional soccer ball, with several changes being made to the shape and design of the surface of the ball. In particular, with the FIFA World Cup tournament in mind, significant changes have been made to the shape and design of the panel of its official soccer ball. The Teamgeist, which was the official ball of the 2006 FIFA World Cup held in Germany, was made of 14 panels, and its form was quite different from the typical form of a conventional soccer ball, which had 32 hexagonal and pentagonal panels. Subsequently, the 8 panel Jabulani (Adidas) was used in the 2010 FIFA World Cup held in South Africa. Later, in the 2013 FIFA Confederations Cup held in Brazil, the 32 panel Cafusa (Adidas) was used as the official ball. The Cafusa had the same number of panels as a conventional ball. However, the panel arrangement of the Cafusa varied depending on the direction of the panel and was quite different from the simple hexagonal and pentagonal panel arrangement of the conventional ball. Then, a ball (Brazuca, Adidas) consisting of 6 panels was used as the official ball in the 2014 FIFA World Cup held in Brazil. The trajectory of this ball was reported to be stable compared to those of other official balls [1, 2]. Earlier, various aerodynamic studies had been conducted on 6, 8, and 14 panel balls as well as the traditional 32 panel soccer balls [3, 4, 5, 6, 7, 8, 9].

**Fig 1:**
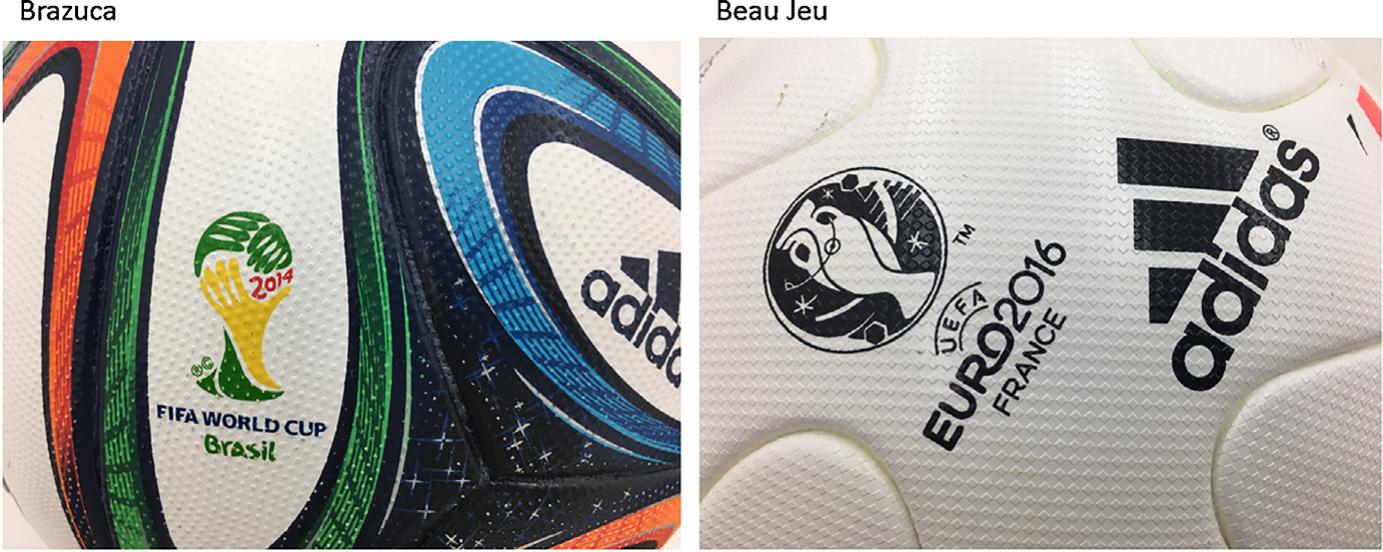
Comparison on the surface form with Brazuca and Beau Jeu. In Brazuca, square projections are spread out giving a wave-like appearance whereas in Beau Jeu, the square projections are arranged uniformly.

The 6 panel Beau Jeu (Adidas), whose panel shape is similar to that of the 6 panel Brazuca but has a slightly different surface form, has been selected as the official ball of the 2016 EURO Cup. The surface of the Beau Jeu has small uneven uniformly arranged square projections; this feature differentiates it from the Brazuca, which has square projections that spread out giving a wave-like appearance (Fig. 1). Considering that changes had so far been made only in the number of panels in the soccer ball, this change in the shape of the ball surface (surface roughness/texture) is believed to be a major evolution. However, the exact impact of this roughness of the ball surface on the aerodynamic characteristics or the flight trajectory of the ball has not been clearly understood.

It is known that a major aerodynamic characteristic of a sports ball is the rapid decrease in the aerodynamic drag when the boundary layer around the ball transitions from laminar flow to turbulent flow during the flight of the ball [10]. The critical Reynolds number has often been considered in earlier studies on the aerodynamic characteristics of a soccer ball [11, 12, 13]. A report on the study of smooth balls shows that the critical Reynolds number is affected by the roughness of the surface of the ball [14]. Further, the form of the seam (such as the depth, width, and length of the seam) has also been said to impact the trajectory of the ball [15, 16]. Furthermore, many aerodynamic studies on footballs have reported that the number, orientation, and shape of the football panels induce a significant effect on the drag characteristics of footballs [1, 2, 12, 17, 18]. However, with new soccer balls of various panel shapes and surface designs being used in official matches in recent years [2, 17], there is a need to clarify their aerodynamic characteristics and critical Reynolds number.

In this study, the pattern of the surface of a soccer ball (surface form including the presence or absence of dimples) is investigated using wind tunnel experiments to determine its impact on the aerodynamic characteristics of the ball. The results confirm that the aerodynamic force acting on the ball vary greatly depending on the impediments on the surface of the soccer ball.

## 2 Methods

### 2.1 Wind tunnel test

**Fig. 2:**
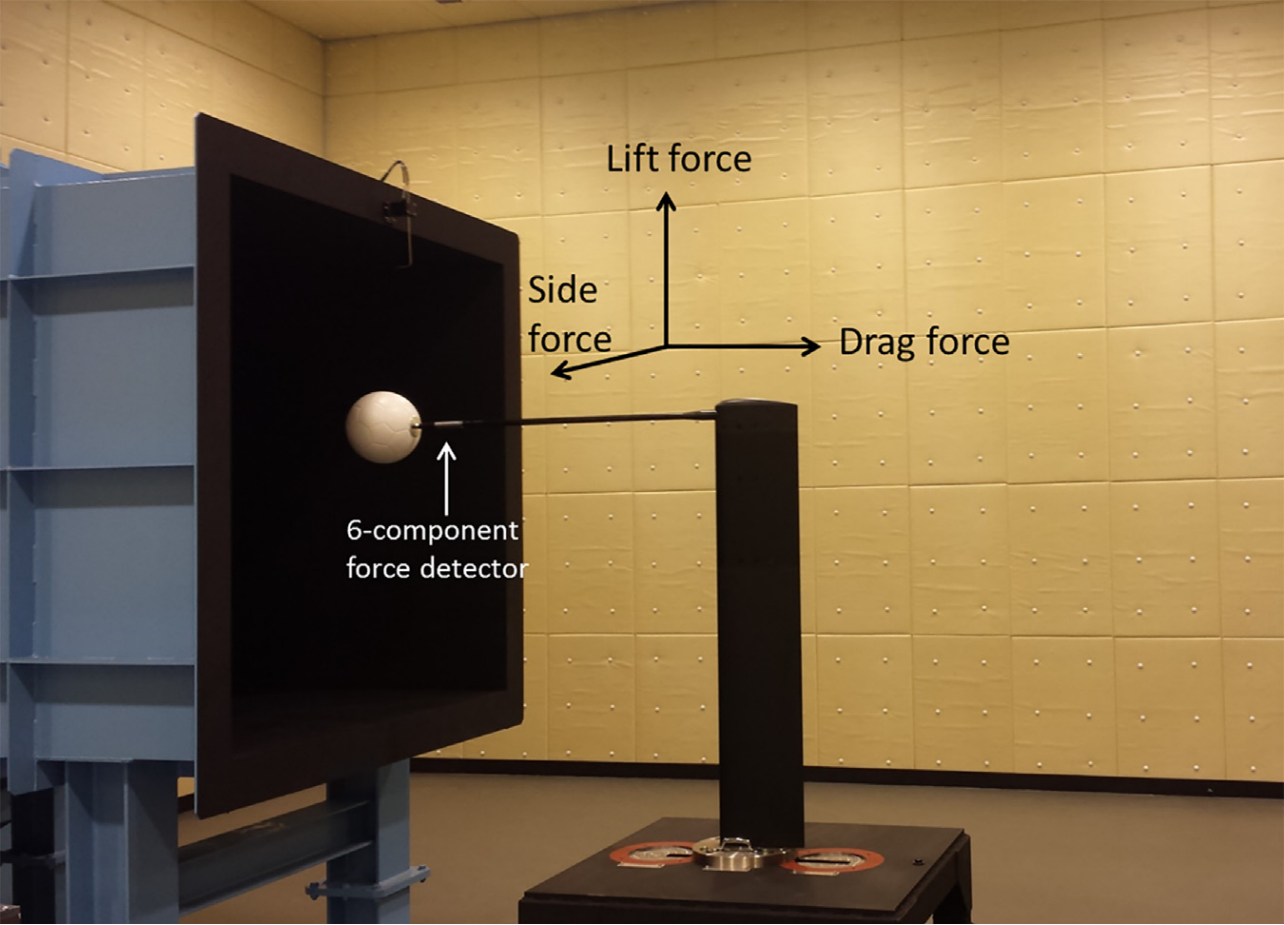
Setup for wind tunnel test.

**Fig. 3:**
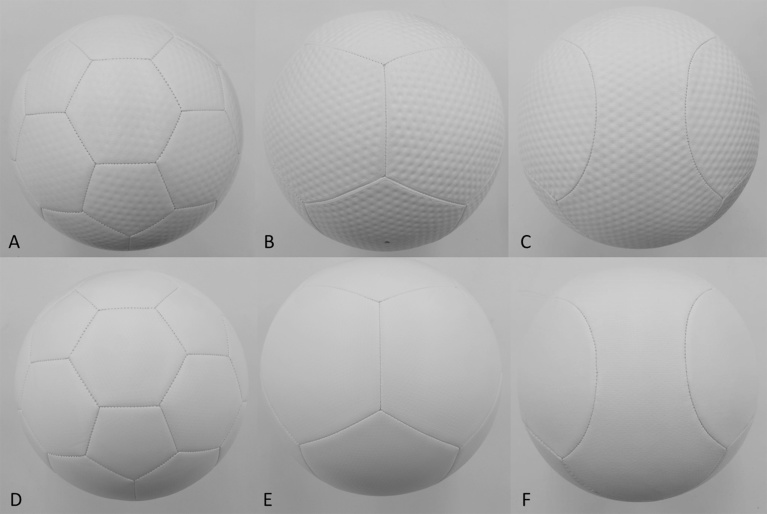
Soccer balls manufactured for the present research: (a) 32_D; dimple-type 32-panel ball, (b) 12_D; dimple-type 12-panel ball, (c) 6_D; dimple-type 6-panel ball, (d) 32_S; dimple-less 32-panel smooth ball, (e) 12_S; dimple-less 12-panel smooth ball, and (f) 6_S; dimple-less 6-panel smooth ball.

The circulating-type low-speed low-turbulence wind tunnel located at the University of Tsukuba (San Technologies Co., LTD.) (Fig. 2) was used in this experiment. The maximum wind speed was 55 m s^−1^, the nozzle size was 1.5 m × 1.5 m, the wind speed distribution was within ±0.5%, the degree of turbulence was less than 0.1%, and the blockage of the measured soccer ball was within 5% of the nozzle size. For example, when the wind speed was set to 25 m s^−1^, the measured mean wind speed was 25.28 m s^−1^, with a standard deviation from the measurement position in the range −0.46 to 0.45 and a wind speed distribution within ±0.5%. Similarly, the degree of turbulence downstream of the nozzle when the wind speed was 25 m s^−1^, was 0.05–0.06, within approximately ±0.1%, so the error from the ball position was believed to have little effect on the wind speed. Furthermore, in the measuring system of this study, the dynamic pressure can be measured automatically with 0.1 kg m^−1^ s^−2^ intervals by means of the Pitot-static tube placed above the measuring portion of the soccer ball. In addition, because the position of the ball during the measurement procedure is set almost at the center of the nozzle cross section to adjust the distance between the nozzle and the ball to zero, the flow generated from the Pitot tube may not have a direct effect on the flow around the ball. Furthermore, the length of the sting used in this study was 0.8 m and its width was 0.02 m. This wind tunnel was used to perform experiments on the same orientation of six type balls. Moreover, because the effect of sting vibrations is reduced to a small value by placing the six-component force detector behind the soccer ball, the magnitude of the sting forces was ignored in this study. We performed experiments on soccer balls made using the same material (leather) in the thread (seam) between the panels. Three types of soccer balls, with 32, 12, and 6 panels, were fabricated, and in each type, balls with and without dimples (width: 3 mm, depth: 1 mm) were made. The diameter of the soccer ball used in this study was uniformly 0.22 m, its weight was 0.436 ± 0.004 kg, and its internal pressure was set to 9.0 psi. In total, six types of soccer balls (Fig. 3) were studied, and the impact of the number of panels, and existence of dimples on the aerodynamic characteristics was measured using wind tunnel experiments. Furthermore, aerodynamic measurements for each soccer ball were performed three times and their average values compared. In this study, aerodynamic measurements for each soccer ball were performed for wind speeds (Reynolds number) from 7 m s^−1^ (approximately *Re* = 1.0 × 10^5^) to 35 m s^−1^ (approximately *Re* = 5.5 × 10^5^) at intervals of 1 m/s.

(1) *Re* =* ν D*/v

Where Reynolds number (*Re*) is a dimensionless number defined as the ratio of inertial force to viscous force, *ν* is wind speed (m s^−1^), *D* is ball diameter (m), and v is the kinematic viscosity (m^2^/s), as shown in Eq. (1).

In the present research, however, a comparative study has been performed by keeping focus on drag coefficient involving simulation of the force acting on the ball due to its surface characteristics.

Also, as wind speed increased, the changes in the side and lift forces on each soccer ball and the corresponding standard deviations were shown for each wind speed and compared, as shown in Eq. (2).

(2) $$SD=\sqrt{\frac{1}{N}\sum_{i=1}^{N}{\left(x_{1}-\mu \right)}^{2}}$$

Where *SD* is standard deviation, ∑ means sum up, *x*_i_ is a value in the data set, *μ* is the mean of the data set, and *N* is the number of data points.

**Fig. 4:**
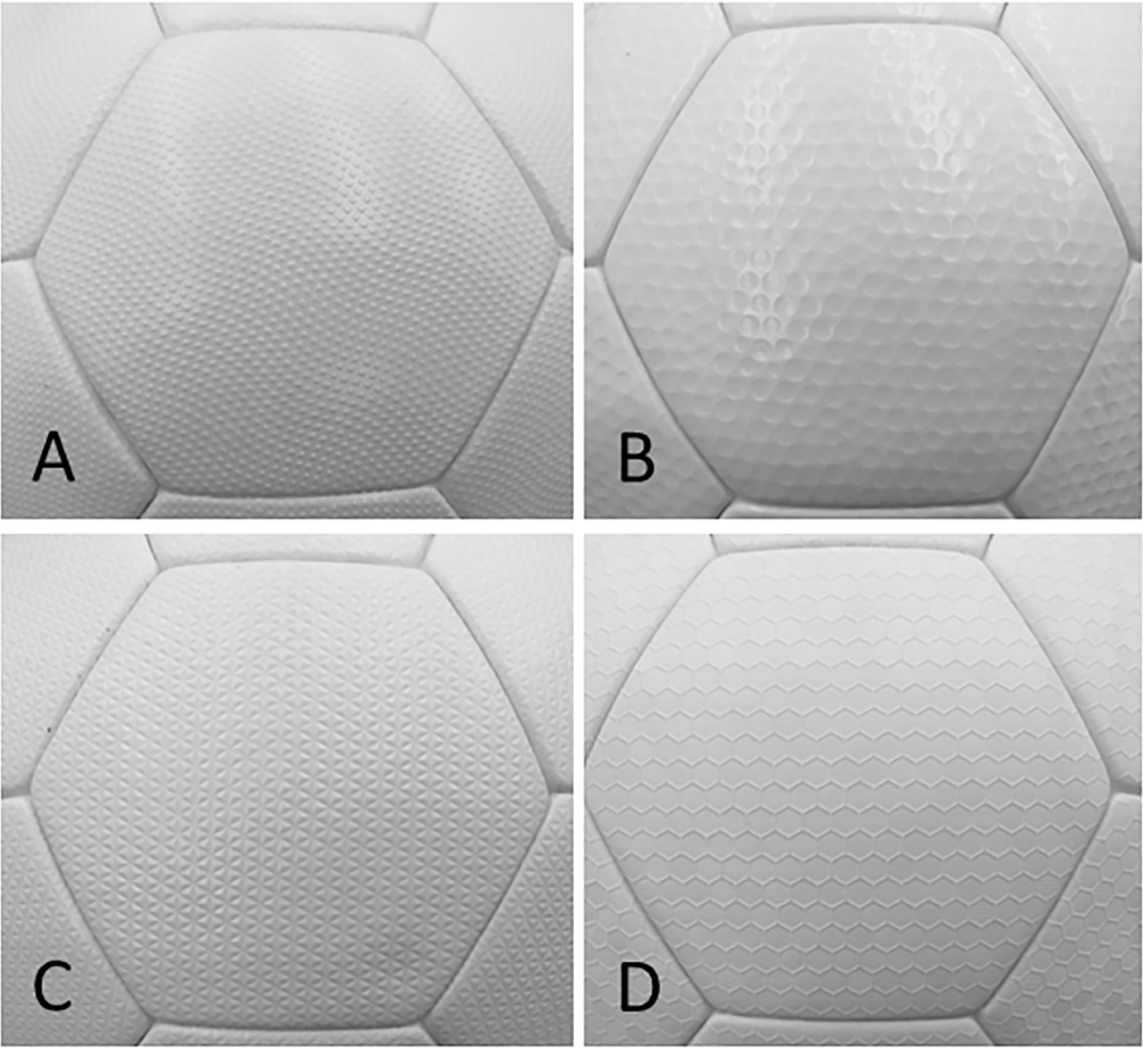
Soccer balls of different surface textures used in the experiment. All the balls had 32 panels. Ball A (32_T1) was made of small square projections in a wave like formation, ball B (32_T2) was made of dimples of 2 different sizes, ball C (32_T3) was made of continuous small triangular projections, and ball D (32_T4) was made of hexagons configured in the form of a honeycomb.

Furthermore, to examine the characteristics of the surface form of soccer balls in greater detail, four types of soccer balls with different surface forms (Fig. 4) were investigated, and a comparative study of the effect of the surface roughness of identical panels (32 pentagonal and hexagonal panels) on the aerodynamic properties was conducted. The panel surface of Type 1 ball (32_T1) was made of small square projections in a wave like formation, whereas that of Type 2 ball (32_T2) was made of dimples of 2 different sizes. The surface of Type 3 ball (32_T3) was made of continuous small triangular projections, whereas that of Type 4 ball (32_T4) was made of hexagons configured in the form of a honeycomb (Table 1). In this study, aerodynamic measurements for four type’s soccer balls were performed for same orientation. Therefore, the six types of soccer balls with or without dimples (Table 1 and Nos. 1–6) can be classified into 3 classes according to panel number (those with 32 panels as a pair, those with 12 panels as a pair, and those with 6 panels as a pair). Measurements were made setting the same panel orientation (Fig. 3) for soccer balls with the same panel number (the pairs). Also, measurements were made setting the same panel orientation (Fig. 4; photograph when seen from the wind tunnel nozzle) for the 32-panel soccer balls with different ball surface textures (Table 1 and Nos. 7–10).

**Table 1:** Types of soccer balls used in this study.

No	Ball name	Ball type
1	32_D	32 panels ball with dimples surface
2	32_S	32 panels ball with smooth surface
3	12_D	12 panels ball with dimples surface
4	12_S	12 panels ball with smooth surface
5	6_D	6 panels ball with dimples surface
6	6_S	6 panels ball with smooth surface
7	32_T1	32 panels ball with T1 surface (small square projections)
8	32_T2	32 panels ball with T2 surface (2 different dimples)
9	32_T3	32 panels ball with T3 surface (small triangular projections)
10	32_T4	32 panels ball with T4 surface (hexagonal projections)
11	1_S	Smooth sphere

**Fig. 5:**
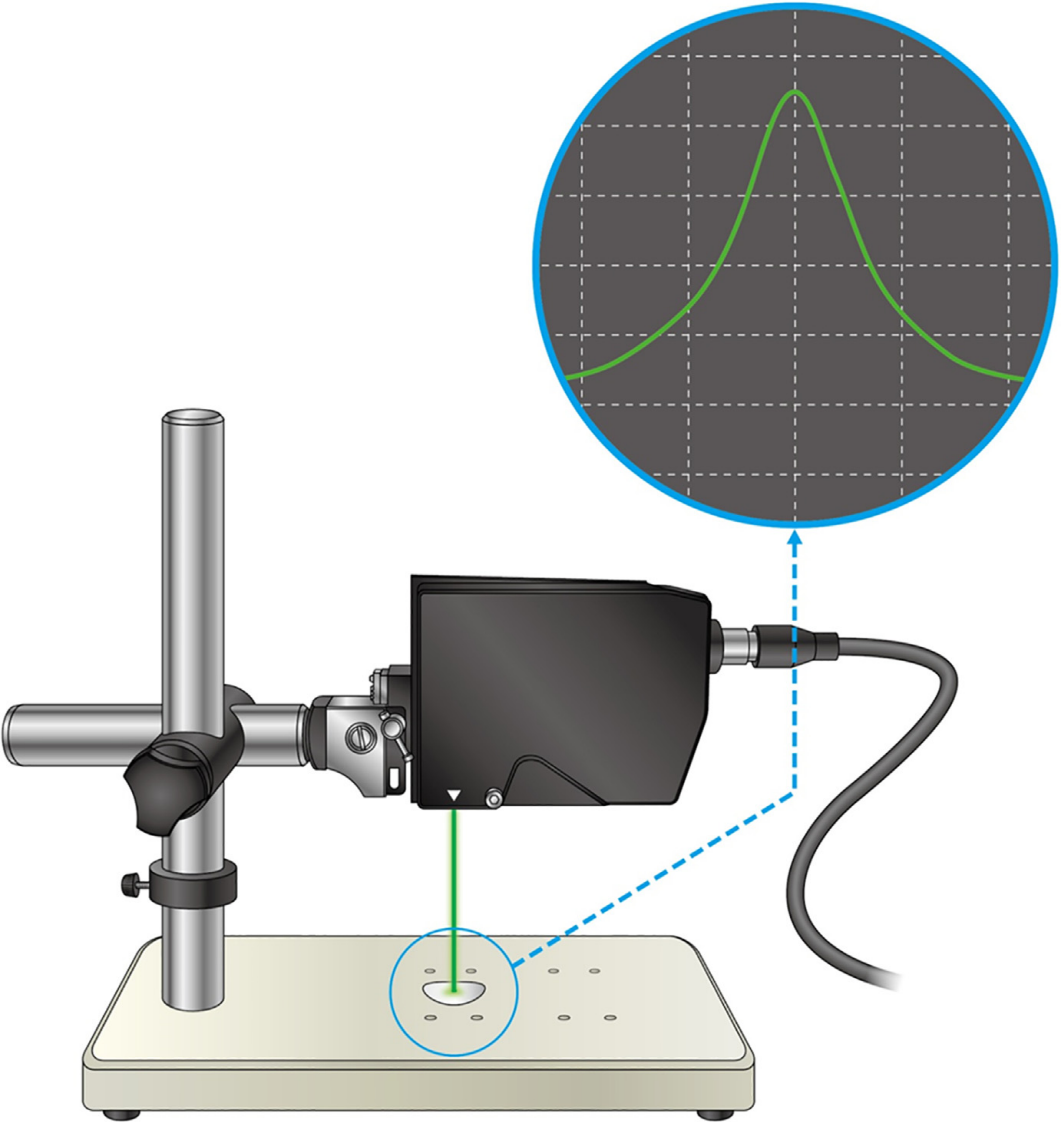
System using a laser scanner to measure the soccer ball surface shape parameters (depth, and width of the panel joints).

The width and depth were measured using a high-speed 2D laser scanner (LJ-V7000, Keyence Corp.) To measure these parameters, all seams of the football were covered using clay with the height of the imprint representing the panel joint depth and the width representing the panel joint width (Fig. 5). The depth and width of the seams of the six types of balls used in this investigation (with and without dimples) are 0.51 ± 0.02 mm and 1.23 ± 0.01 mm, respectively, and those of the four types of soccer balls made with 32 panels were 0.92 ± 0.03 mm and 3.34 ± 0.05 mm, respectively.

The force acting on the soccer ball was measured by using a sting-type 6-component force detector (LMC-61256, Nissho Electric Works). The measured aerodynamic force was converted into the drag coefficient (*C_d_*), lift coefficient (*Cl*), and side coefficient (*Cs*), as shown in Eqs. (3)–(5), respectively.

(3) $$C_{d}=\frac{2D}{\rho U^{2}A}$$

(4) $$C_{l}=\frac{2L}{\rho U^{2}A}$$

(5) $$C_{s}=\frac{2S}{\rho U^{2}A}$$

Where *ρ* is the air density (*ρ* = 1.2 kg m^−3^), *U* is the wind speed, and *A* is the projected area of the football (*A* = *π* × (0.11)^2^ = 0.038 m^2^).

### 2.2 Ball trajectory simulations

We conducted a simple 2D flight simulation to compare the effects of the drag coefficients of the 12 panel dimple-type ball (12_D) and the 12 panel dimple-less ball (12_S) in the super-critical regime (*Re* = 4.6 × 10^5^) and the 32 panel dimple-type ball (32_D) and the 32 panel dimple-less ball (32_S) in the critical regime (*Re* = 2.3 × 10^5^) on their flight distance and flight trajectory [13]. The occurrence of irregular and unsteady lift and side forces known as the ‘knuckle effects' has been reported for soccer balls flying with no spin or a low-speed spin [7, 8, 19]. However, because this study focused on the relationship between the constant resistance of the ball and its flight trajectory [12, 18], knuckle effects were ignored in the trajectory simulation. In this trajectory simulation, we estimated the drag coefficient with respect to the Reynolds number using a cubic curve to calculate the two-dimensional coordinates of the ball. Therefore, we omitted the lift and side forces acting on the ball. We considered the knuckle effect of on the flight trajectory to be negligible compared with the effect of drag; therefore, we omitted the knuckle effect from our calculations.

According to Newton's second law, the simulation calculation is as shown in Eq. (6).

(6) $$m\vec{a}=\vec{F}$$

Where *m* is the mass of the soccer ball, $\vec{a}$ is the acceleration, and $\vec{F}$ is the force (force acting on the ball + gravitational force).

In flight simulation, by using the *Cd* value and Reynolds number obtained from wind tunnel experiments, comparison was made at every interval (critical regime and supercritical number) by selecting conditions such that the difference between the flight distance of the two balls with and without dimples is a maximum. Thus, by setting various conditions, the difference between the flight distances of various balls can be explained with more clarity.

## 3 Results and discussion

### 3.1 Aerodynamic effect of the presences of dimples on soccer ball surfaces

**Fig. 6:**
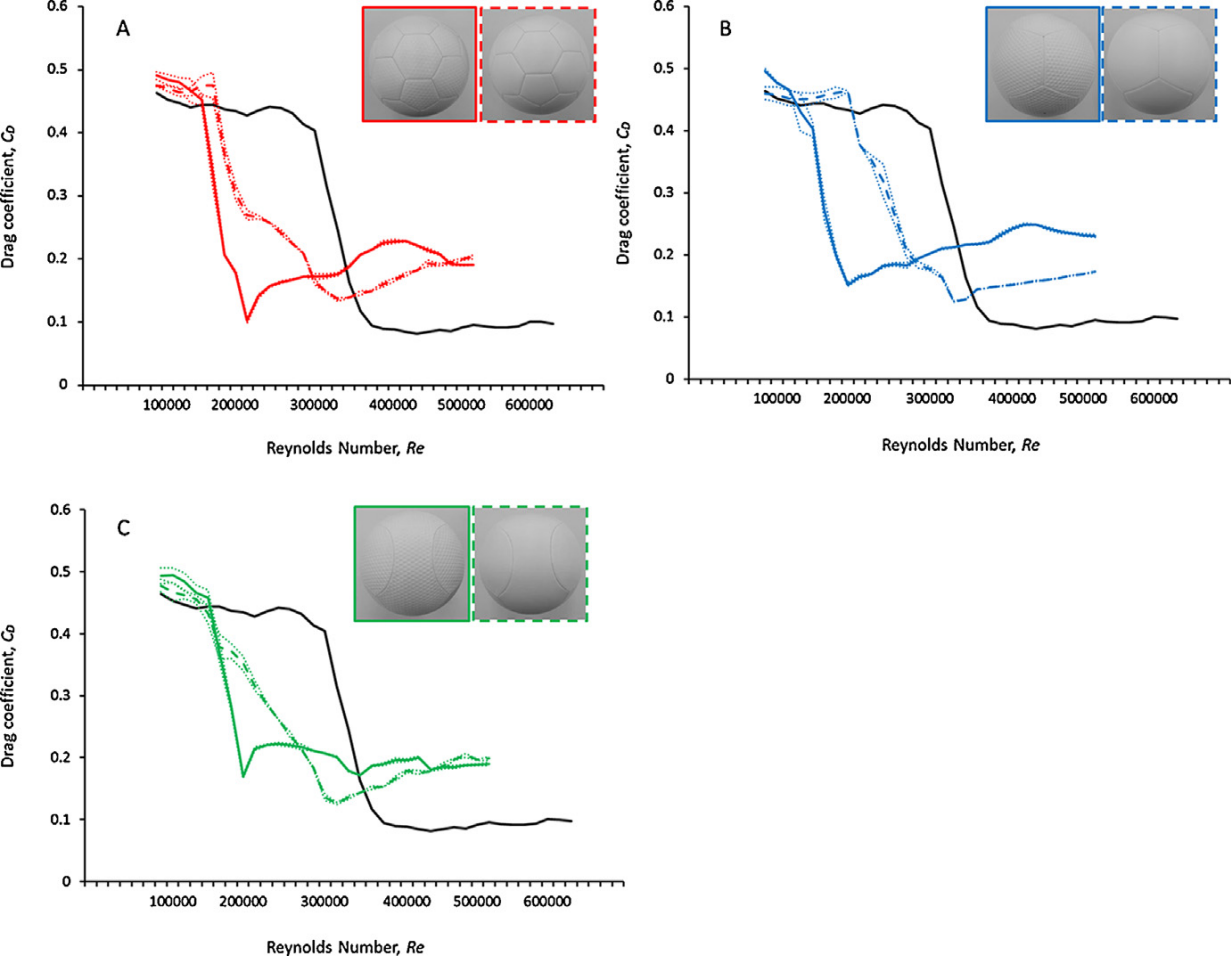
Drag coefficient (*C_d_*) of the soccer balls. Parts A, B, and C represent the balls consisting of 32 panels (red line), 12 panels (blue line), and 6 panels (green line), respectively. The solid line represents the ball with dimples, whereas the dashed line represents the ball without dimples. And the dotted line indicates the standard deviation for each ball.

The aerodynamic coefficients of the balls indicate that the aerodynamic drag (Fig. 6) tends to drop faster for the dimple-type soccer balls (solid lines) than for the dimple-less soccer balls (dashed lines). Furthermore, in soccer balls with 12 and 6 panels the *Cd* value for balls without dimples showed a trend of being smaller than that for balls with dimples in the supercritical range (Fig. 6B and C). However, for 32-panel soccer balls in the supercritical range, the dimple-less type had a lower *Cd* value than the dimple-type ball (Fig. 6A). This *Cd* value indicates the average for various balls and the Pearson product-moment correlation coefficient for practice results was *r* = 0.95 (*p* < 0.01); therefore, the difference caused by practice could not be seen. This is believed to be due to the fact that in the critical Reynolds number range (*Re* = 1.5 × 10^5^–3.0 × 10^5^), the dimple-type soccer ball experiences a smaller drag (resistance) than the dimple-less ball. From the results, in the case of dimple-type balls, in the critical Reynolds number range, the shape of the dimples causes transition of the boundary layer on the ball. Therefore, the dimple-type balls are perceived to be faster than the dimple-less balls. In contrast, in the supercritical Reynolds number range (*Re* = 3.8 × 10^5^–5.0 × 10^5^), the dimple-less ball had a smaller drag value. Furthermore, it can be inferred that frictional drag for dimple-less balls are lower than that for the dimple type ball in the supercritical Reynolds number range. Therefore, we found that the drag acting on the ball changes depending on the Reynolds number interval. The 32_D soccer ball had the smallest supercritical drag coefficient of approximately 0.10 (*Re* = 2.3 × 10^5^), and the 12_D ball had a corresponding value of approximately 0.15 (*Re* = 2.1 × 10^5^). The 6_D soccer ball had a corresponding value of approximately 0.17 (*Re* = 2.1 × 10^5^). Further, in the case of the dimple-less balls, the value for the 32 panel ball was approximately 0.13 (*Re* = 3.5 × 10^5^) and that for the 12 panel ball was approximately 0.12 (*Re* = 3.5 × 10^5^). The 6 panel ball had a value of approximately 0.12 (*Re* = 3.4 × 10^5^). In the case of the dimple-type balls, the supercritical drag coefficient increased as the number of panels reduced, but no such difference attributable to the number of panels was observed in the case of the dimple-less balls. For soccer balls with dimples, the supercritical drag coefficient was observed to increase as the number of panels decreased, so it is believed that the supercritical drag coefficient will decrease as the total length of the seams on the surface of the soccer balls with dimples increases (total length of the seams increases as panel number increases). Furthermore, we observed the same trend as reported in prior research: that the length of the seams has a large effect on the supercritical drag coefficient [6]. While a correlation was not observed between the total length of the seam and the supercritical drag coefficient in dimple-less balls, dimple-less balls with 12 or 6 panels had a lower supercritical drag coefficient than dimple-type balls with the same number of panels. Further, this result is believed to be the same as in prior research [14]. For the 32-panel type, however, the dimple-type ball had a slightly lower value. This suggests that while the panel number has little effect on the supercritical drag coefficient in dimple-less balls (sequentially, 0.13 for the 32-panel ball, 0.12 for the 12-panel ball, and 0.12 for the 6 panel ball), dimples in the surface of a ball has a large effect on its supercritical drag coefficient. Therefore, the effect of the dimples is believed to be relatively larger than the effect of the total length of the seams. However, it is necessary to study these phenomena in more detail in the future.

From these results, the dimple shape in the soccer ball surface is also expected to have the effect of maintaining a constant boundary layer around the ball. However, because the air flow around the ball was not studied in this experiment, it is necessary to study from multiple angles how friction resistance, due to the presence or absence of dimples, affects boundary layer separation, so in the future, it will be necessary to study the ball surface air flow in further detail by using visualization methods such as PIV.

**Fig. 7:**
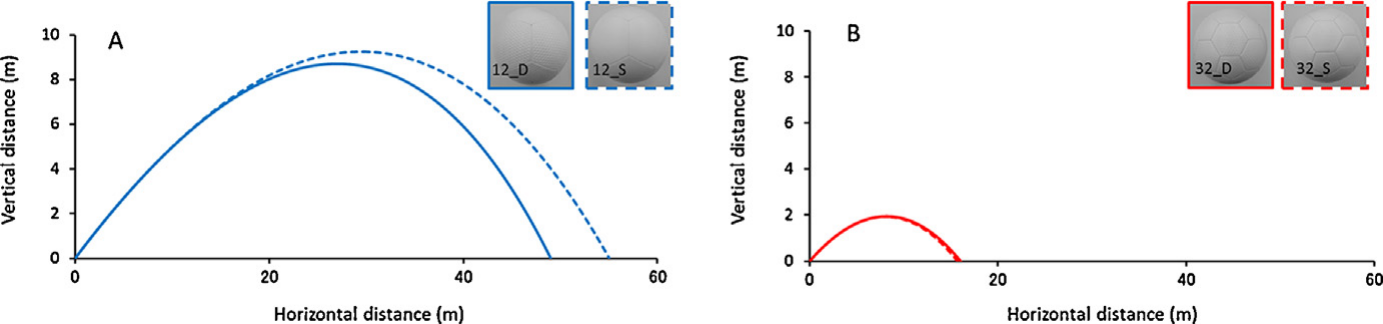
Comparison on the flight distance of soccer balls (A: ball initial velocity: 30 m s^−1^, attack angle: 30°, B: ball initial velocity: 15 m s^−1^, attack angle: 25°).

**Fig. 8:**
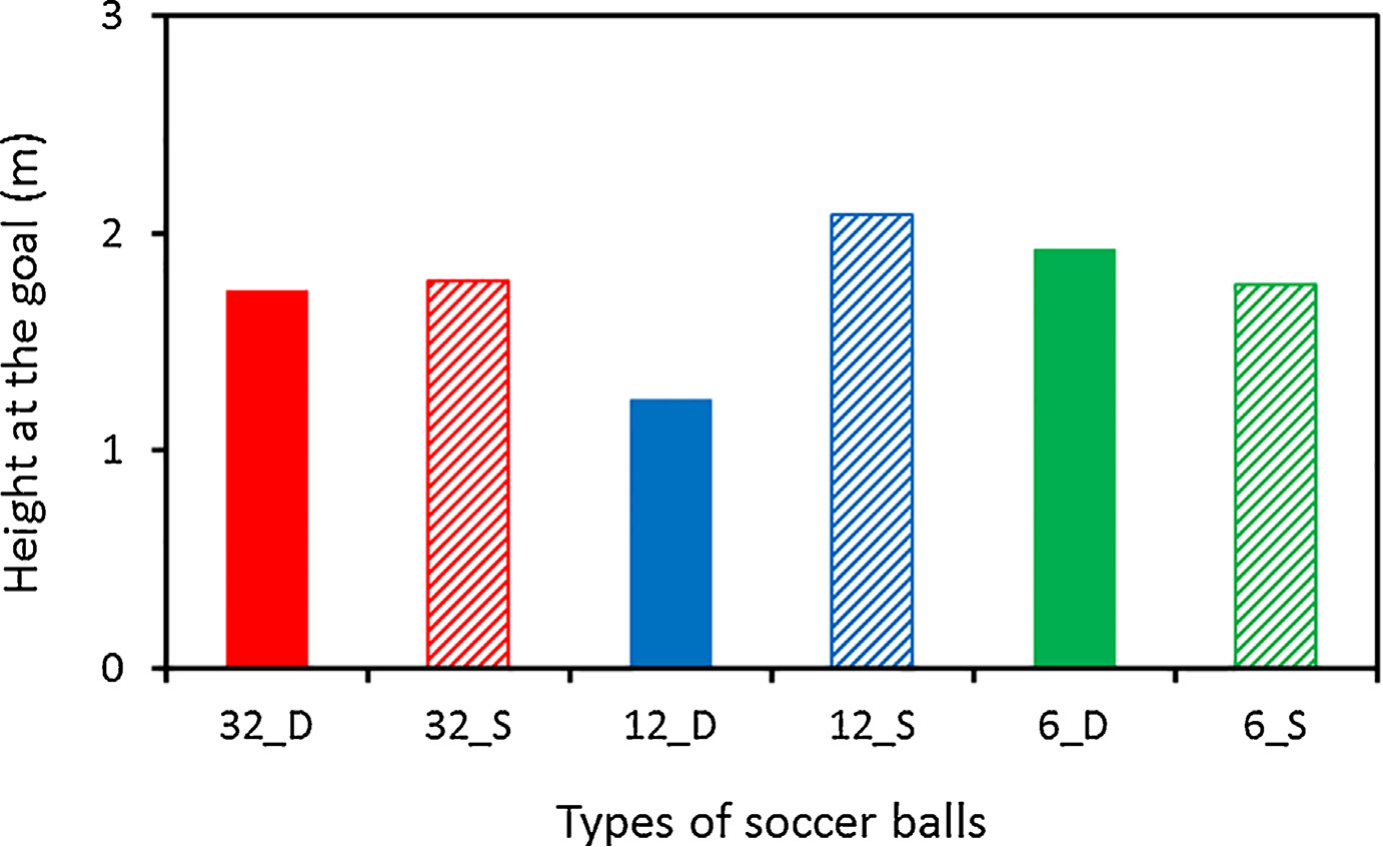
Locations where the six balls would cross the goal plane (*v_0_* = 30 m s^−1^ and *θ_0_* = 18°).

Using a ball trajectory simulation in the supercritical regime (ball initial velocity: 30 m s^−1^, attack angle: 30°), we compared the flight distance of the 12_S ball (a small drag coefficient), and the 12_D ball (a large one). The flight distances for the 12_S ball and 12_D ball were approximately 55.1 and 49.0 m respectively (Fig. 7A). In the same manner, using a simulation of a ball flight in the critical regime (ball initial velocity: 15 m s^−1^, attack angle: 25°), comparing the flight distance of the 32_D ball and the flight distance of the 32_S ball, the 32_D ball distance (approximately 17.9 m) was approximately 0.5 m longer than that of 32_S ball (approximately 17.4 m) (Fig. 7B). Further, Fig. 8 shows where each of the six balls passes through the goal plane. We model a long FK (free kick) for which the ball was placed on the pitch 35 m away from the center of the goal. Each ball is kicked no spin at *v_0_* = 30 m s^−1^ and *θ_0_* = 18°. The impact point of the 12_S ball (approximately 2.1 m) was approximately 0.4 m higher than that of 12_D ball (approximately 1.7 m). However, in the present study, investigation of the unsteady aerodynamic force acting on the soccer ball in air has not been performed (such as knuckle effect); therefore, in future research it is necessary to investigate the difference in the knuckle effects of soccer balls in-flight by studying the vortex structure of the wake flow of the soccer ball in flight.

**Fig. 9:**
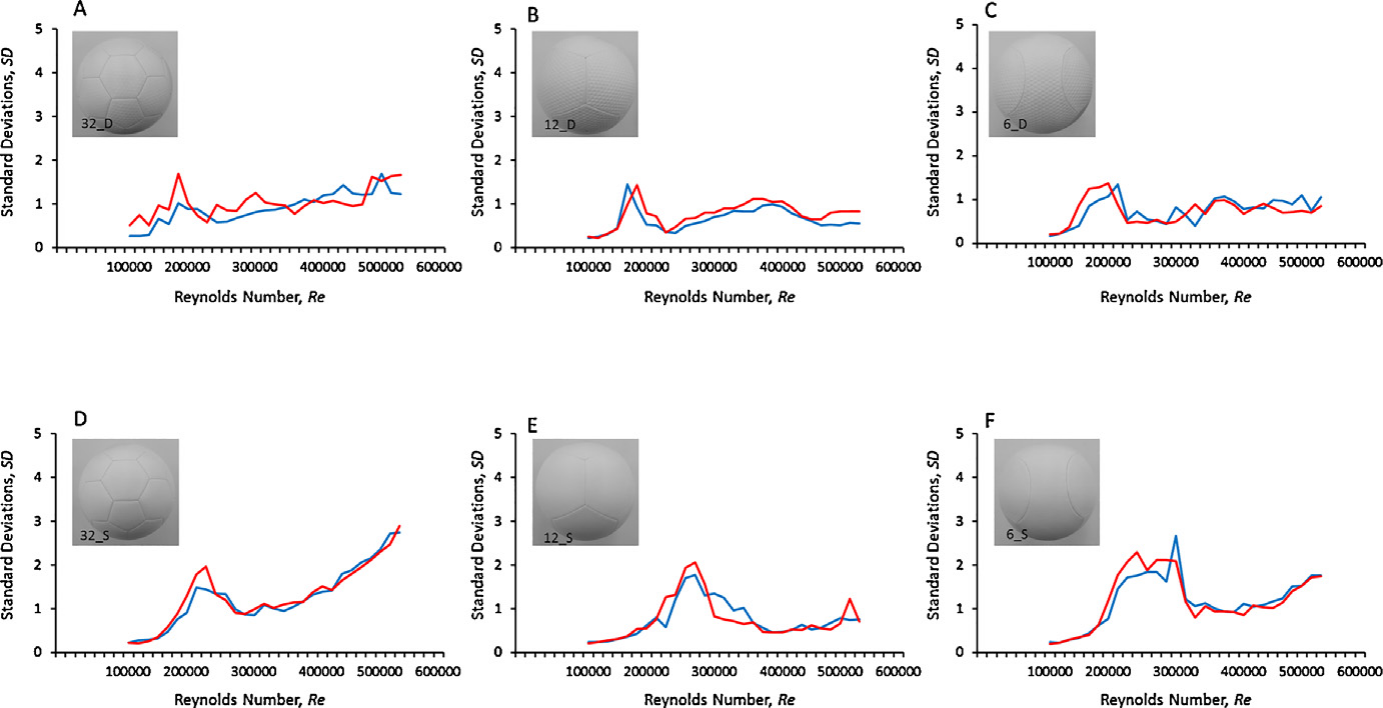
Variation in lift and side coefficients due to the Reynolds number. (blue line: side coefficient, red line: lift coefficient). Parts A and D, B and E, and C and F represent balls with 32, 12, and 6 panels, respectively. Parts A, B, and C represent balls with dimples, whereas parts D, E, and F represent balls without dimples.

Fig. 9 shows the graph of the force variation (standard deviation (*SD*)) due to the increase in the flow velocity for the six types of soccer balls divided according to the presence or absence of dimples and the number of panels. First, looking at the entire graph, the lift coefficient (red line) and side coefficient (blue line) acting on the ball are seen to vary greatly depending on the types of ball. Further, although the force variation is different for the ball types, no significant difference were observed in the tendency for force variation in the lift and side coefficients in the same type of balls. In terms of the force variation (*SD*) in the side and lift coefficients due to the presence or absence of the dimples, the dimple-less balls (Fig. 9D, E and F) are seen to have a larger value than the dimple-type balls (Fig. 9A–C) with an increase the flow velocity. In particular, for the 32_S ball (Fig. 9D), at flow velocities more than 30 m s^−1^ (*Re* = 4.6 × 10^5^), the variation is seen to exceed 2 in terms of both the lift and side coefficients, showing a greater variation than that for the other types of balls with an increase the flow velocity. However, for the 32_D ball, the magnitude of the force variation is seen to be less than 2, showing a smaller variation than that for the dimple-less ball (Fig. 9A). For example, in the case of the flow velocity at 30 m s^−1^ (*Re* = 4.6 × 10^5^), the variation in the lift and side coefficients of the 32_D ball was approximately 1.2 and 0.9 (respectively). However, the 32_S ball was approximately 2.0 and 1.9 (respectively). Therefore, the dimple-type balls are observed to have less force variation than the dimple-less balls. These results indicate that the shape of dimples on the surface of the soccer ball may have the effect of constantly maintaining a boundary layer around the ball. Moreover, it has been made clear that the dimple structure on the surface of the soccer ball significantly affects the force variation on the flight distance and force variation on lift force and side force according to the Reynolds number intervals. Therefore, we found that the drag acting on the ball changes depending on the Reynolds number interval. Furthermore, it is now understood that the aerodynamics of the ball varies not only because of the structure of the dimples, but also because of the shape of the surface protrusions. Also, the surface of the ball involves complicated factors such as shape or number of panels, their width and depth and therefore, a detailed study of the flow of air at the surface of the ball by using a visualization method such as PIV is essential in future studies.

### 3.2 Effect of different soccer ball surface shapes on aerodynamic characteristics

**Fig. 10:**
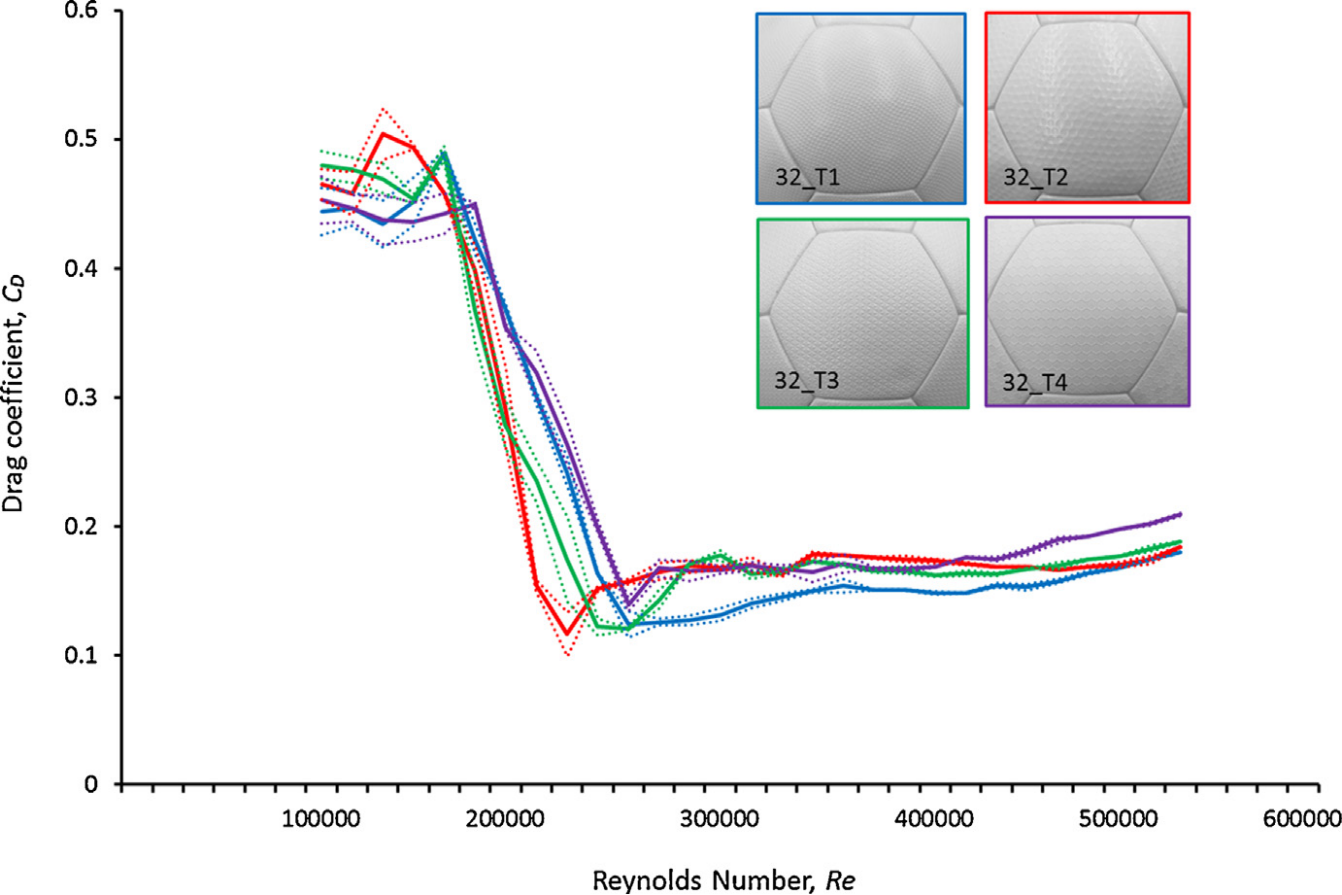
Change in *Cd* for the four types of soccer balls having different surface forms. Panel surfaces denoted as Types 1, 2, 3, and 4 have small square projections, dimples, triangular projections, and hexagonal projections, respectively. The solid line indicates the average values for various balls and the dotted line indicates the standard deviation for each ball.

**Fig. 11:**
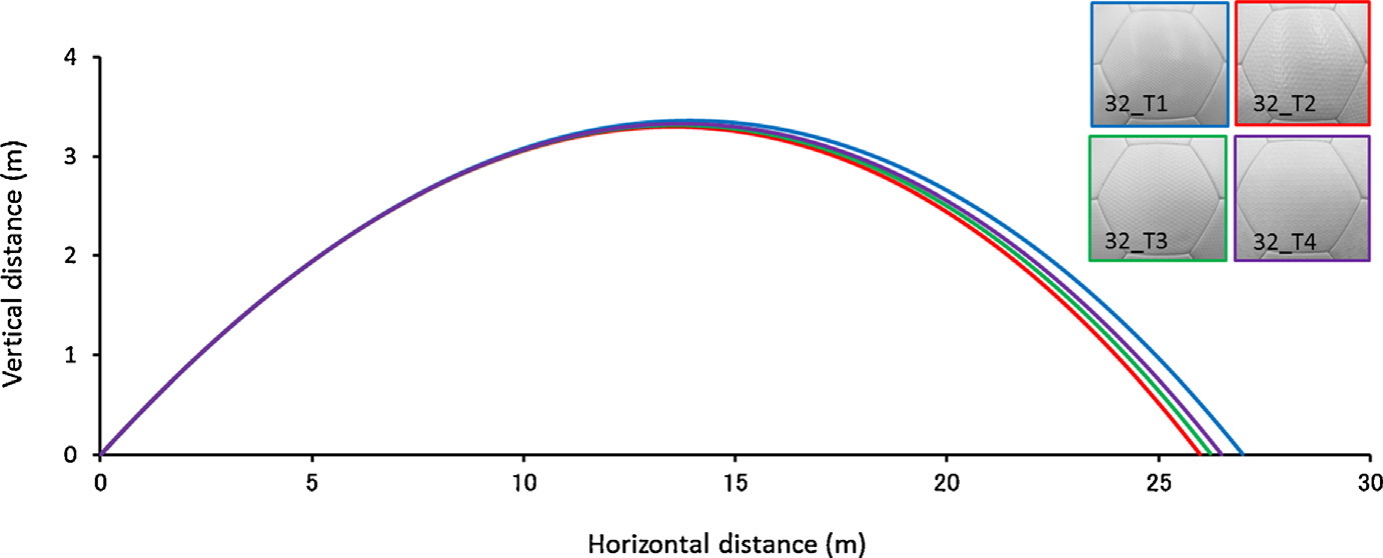
Comparison on the flight distance of four type soccer balls (*v_0_* = 20 m s^−1^ and *θ_0_* = 18°).

Fig. 10 is a graph that contains the change in *Cd* values in the four types of soccer balls with different surface shapes. Each of these four types of soccer balls consists of 32 same panels. Also, the measured ball orientation was set identically to that shown in Fig. 4 in order to perform measurement. First, the drag coefficients of the balls indicate that the 32_T2 ball with dimples tends to fall more quickly compared to the other types of balls. This result was similar to the results of Fig. 6 in terms of the presence of dimples and shows that in the critical Reynolds number range (*Re* = 1.5 × 10^5^–3.0 × 10^5^), the dimple-type balls have a smaller drag (resistance) than the balls with other types of panels. In addition, the 32_T1 ball experiences a smaller drag than the other balls in the supercritical Reynolds number range used during a long kick or a powerful shot (*Re* = 3.8 × 10^5^–5.0 × 10^5^). Further, the 32_T2 soccer balls with dimples have the smallest supercritical drag coefficient of approximately 0.11 (*Re* = 2.3 × 10^5^), whereas the 32_T4 balls with hexagonal surface forms have the largest value of approximately 0.14 (*Re* = 2.7 × 10^5^). Moreover, the 32_T1 balls made of square projections have a value of approximately 0.12 (*Re* = 2.7 × 10^5^), whereas the 32_T3 balls with triangular projections have a value of approximately 0.12 (*Re* = 2.7 × 10^5^). Further, we compared the flight distance of these four different types using a ball trajectory simulation in the supercritical regime (ball initial velocity: 20 m s^−1^, attack angle: 18°). The flight distances for the 32_T1 ball and 32_T2 ball were approximately 27.0 and 25.9 m respectively (Fig. 11). Therefore, the dimple-type ball (32_T2) experiences smaller air resistance in the critical Reynolds number range than the balls with other surface forms. Hence, the 32_T2 ball is perceived to be faster than the other types of balls in the critical Reynolds number range. However, in this study, basic investigation of how general characteristics such as the presence or absence of dimples and the pattern of their protrusion affect the soccer ball aerodynamics was conducted. Consequently, the relationship between the shape of various dimples and the aerodynamics of the ball is a topic for future research.

**Fig. 12:**
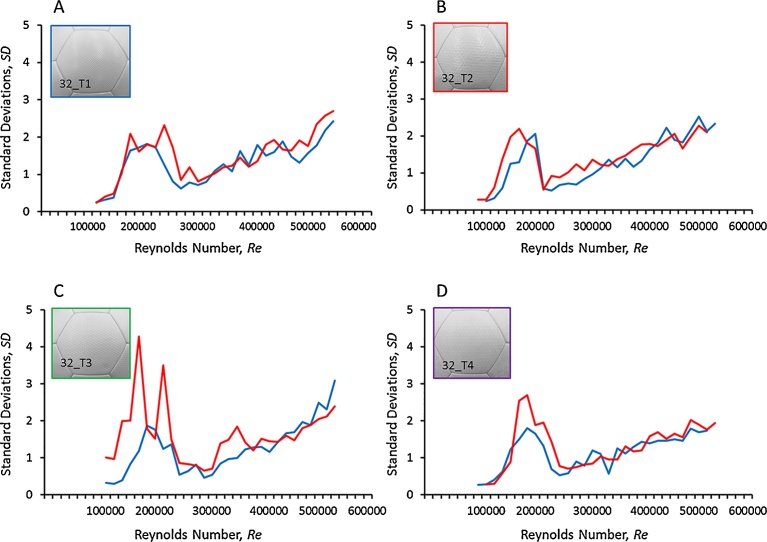
Changes in lift and side coefficients due to differences in the roughness of the soccer ball (blue line: side coefficient, red line: lift coefficient). Graph A shows the results for type 1 ball (32_T1), and graphs B, C, and D show the results for types 2, 3 and 4 (32_T2, 32_T3, and 32_T4), respectively.

Fig. 12 shows the graph of the force variation in the side coefficient and lift coefficient due to an increase at flow velocity for four soccer balls with four different surface forms. Small changes are observed in the force variation in the lift and side coefficients for each panel type. However, the 32_T4 ball with a hexagonal pattern on its surface (Fig. 12D) has a smaller variation in the lift and side coefficients than the other three types of balls. Considering the variation in lift and side coefficients in the speed interval used for a powerful shot in particular (*Re* = 4.2 × 10^5^), the 32_T4 ball has values of approximately 1.45 and 1.50 (respectively), which are smaller than the corresponding values for the other types (32_T1 ball: 1.89 and 1.67, 32_T2 ball: 2.22 and 1.73, 32_T3 ball: 1.66 and 1.59, respectively). It is seen that dimples (surface texture) have a greater impact on the aerodynamic characteristics of the balls. Further, creating forms on the surface of the soccer ball makes it possible to control the irregular movement of the ball in the up and down and left and right directions to some extent. Thus, the fact that the aerodynamic force acting on the ball varies depending on the surface form of the soccer ball suggests that surface roughness is also one of the important factors that determine the aerodynamic characteristics of the ball in addition to the shape and number of panels.

## 4 Conclusions

Recent studies on the fluid dynamics of soccer balls have reported that the number of panels on the ball, their orientation, and changes in panel shapes have a major impact on the aerodynamic characteristics of the ball. The present researches also show results similar to those of previous studies. In particular, this study has established the fact that changing the texture of the ball surface changes the aerodynamic characteristics of the ball. This suggests that by considering the changes in the aerodynamic characteristics obtained in response to variations in the number and shape of the panels and modifications to the surface form, soccer balls with even more diverse aerodynamic characteristics can be developed. However, it is necessary to examine dimples of various sizes and patterns other than the ones used in this study. Therefore, in the future, we intend to study the surface roughness in more detail by examining the air flow around the dimple on the ball surface through visualization experiments such as particle image velocimetry.

## Declarations

### Author contribution statement

Sungchan Hong and Takeshi Asai: Conceived and designed the experiments; Performed the experiments; Analyzed and interpreted the data; Contributed reagents, materials, analysis tools or data; Wrote the paper

### Competing interest statement

The authors declare no conflict of interest.

### Funding statement

This work was supported by JSPS KAKENHI Grant Number 24240084, 25750283, 15K16442 of the Ministry of Education, Culture, Sports, Science and Technology of the Japanese government.

### Additional information

No additional information is available for this paper.
